# Rosin-Weed—Silphium Laciniatum

**Published:** 1868-01

**Authors:** H. D. Garrison


### ROSIN-WEED—SILPHIUM LACINIATUM.

A NEW REMEDY; REPORTED A SPECIFIC IN ASTHMA.

By H. D. GARRISON, M.D.,

Within the last eighteen months, several persons have, in a more or less confidential manner, informed me, that the rosin-weed is an infallible remedy for asthma. Numerous parties, professional and non-professional have related to me cures effected by this agent in cases before considered wholly incurable by the medical faculty.

The substance of this information, derived thus from parties in most cases entirely unaware of the existence of any previous information on the subject, as they were in some cases even ignorant of the existence of each other, not only makes the discovery justly common property, but gives it a degree of reliability not pertaining to the reputation of many new remedies.

I would not willingly assist in placing another unnecessary or doubtful remedy on the already overburthened catalogue; but were it possible would with pleasure aid in displacing a legion that only confuse and deceive the practitioner, and encumber the materia medica.

Farmers and others, intelligent on equine hygiene and treatment, quite uniformly agree that this plant is not only a perfect preventive, but a specific remedy for the heaves of horses, which is essentially the same disease as asthma in man.

Dr. G. H. Dadd of this city, in his new and very able work, entitled "*American Horse and Cattle Doctor*," page 125, says, "the husbandmen who reside in the vicinity of where rosin-weed grows, are well acquainted with the properties of this plant, and they declare that it is a specific for the treatment of asthma, or heaves. I have used the article in the form of fluid extract prepared from the root, and find it to be a very valuable remedy. The dose (for a horse) of the fluid extract is two (fluid) ounces, morning and evening." After describing some varieties of asthma or heaves, necessarily incurable, because of some lesion of the respiratory apparatus, Dr. Dadd further adds, that "such cases, although considered incurable, may be palliated by the fluid extract of rosin-weed." In a recent conversation with Dr. Dadd, he declared himself, more than ever, convinced of the great efficacy of this plant in the cure of *asthma in man* as well as in horses. It is singularly corroborative of the foregoing views that in those prairie regions where the rosin-weed abounds, also in those cities whose markets are supplied with hay from such sources, asthma or heaves in horses is quite unknown. During my residence in this city I do not recollect seeing a single case of heaves, though the streets are alive with horses which are not better used or fed than their race in the Middle and Eastern States where no affection is more common among them. Horses like this plant very much. On being fed hay containing it, they select all the rosin-weeds and eat them first.

Prof. King (Am. Dis. p. 871), ascribes to the *silphium perfoliatum* tonic, diaphoretic, and alterative properties, and alludes to its successful employment in ague-cake (enlarged spleen), liver complaints, miasmatic fevers, etc. In the same article, under the head *Silphium Laciniatum* and *S. Gumiferum*, in addition recommends them in "dry, obstinate coughs," and laconically adds, "said to cure the heaves in horses."

To the foregoing therapeutic properties, should they be established by more extended experience, may undoubtedly be added another, viz.: *powerfully diuretic.* A very intelligent gentleman, who has collected large quantities of this and other medicinal plants for us during the past two years, stated to the writer, a few days since, that the rosin-weed acts so powerfully on the kidneys as to cause dull pain, and even considerable distress in the region of the kidneys of those who used it to any great extent. This property, mentioned also by others, will suggest to the intelligent physician a much wider range of usefulness than has before been ascribed to this plant. There are many diseases of the urinary apparatus yet deemed incurable, while most of such affections are by no means uniform in their submission to treatment. It is hoped that this remedy will supply some deficiency in the list of real remedies.

The rosin-weed, *silphium luciniatum*, known also by the common names "polar plant" or "compass plant," from the circumstance that its leaves quite uniformly point North and South, is a member of the large order *Compositæ*, and abounds throughout the high rolling Western prairies. The stem is from three to ten feet high, and rough, with white hispid hairs. Leaves are one-half to two feet long, much divided, alternate and lower ones petiolate. The stalk bears four to eight large showy heads with yellow rays, and flowers in July and September. The root is large, sprangling, and of a tough, leatherly consistency. A smoothly cut surface presents a resinous lustre. The entire plant is possessed of a bitterish taste, but pleasant aroma, due to a volatile oil. Other species of this genus, as *S. Perfoliatum*, are also known as "rosin-weed," "Indian cup plant," and are possessed of similar, if not identical, medicinal properties. The whole genus, embracing five species, abounds in volatile oil and resin, which exudes in the form of small white spots or tears similar to those of mastich.

I have made a thorough chemical investigation of this plant to determine in what form its active principles could best be presented for administration; and conclude, that a fluid extract of high alcoholic strength is the only reliable form in which it can be used. The root when fresh is rich in essential oil, which is gradually converted by oxidation into resin, just as oil of turpentine by oxidation produces common rosin. As the resin of the silphium is not pulverizable, and as it is not yet known whether the good effects of the remedy are due to the oil or to the product of its oxidation (resin), it is plain that no "powder" can fully represent the plant. A solid extract from which most of the oil is necessarily absent would not be eligible unless the activity is first shown to reside in the resin or some other part of the plant besides the oil.

Infusions of decoctions or oleiferous articles, though often the only form at command, are never more than fractional representations of the articles employed. The powdered root or plant would contain but little oil, owing to the thorough drying necessary to effect pulverization.

A fluid extract skilfully made as above indicated, will contain all the oil, resin, and every other medicinal proximate principle of the plant in their native state and proportions, together with all the freshness and aroma of the plant unimpaired, and will accurately represent the root, drop for grain. The dose of the fluid extract is from twenty to forty drops.—*Am. Ec. Med. Rev.*

